# Neuroprotective effect of docosahexaenoic acid nanoemulsion on erectile function in a rat model of bilateral cavernous nerve injury

**DOI:** 10.1038/srep33040

**Published:** 2016-09-14

**Authors:** Chun-Hou Liao, Yi-No Wu, Bin-Huei Chen, Ying-Hung Lin, Hsiu-O Ho, Han-Sun Chiang

**Affiliations:** 1 Division of Urology, Department of Surgery, Cardinal Tien Hospital, Taipei City, Taiwan; 2 School of Medicine, Fu Jen Catholic University, New Taipei City, Taiwan; 3 PhD Program in Nutrition & Food science, Fu Jen Catholic University, New Taipei City, Taiwan; 4 Graduate Institute of Biomedical and Pharmaceutical Science, Fu Jen Catholic University, New Taipei City, Taiwan; 5 Department of Food Science, Fu Jen Catholic University, New Taipei City, Taiwan; 6 School of Pharmacy, Taipei Medical University, Taipei City, Taiwan

## Abstract

There is an unmet need for treatment of erectile dysfunction resulting from radical prostatectomy and cavernous nerve (CN) injury. Given the neuroprotective properties of docosahexaenoic acid (DHA), we investigated its effect on penile functional and structural recovery in a rat model of bilateral cavernous nerve injury. Rats were subject to CN injury and received intraperitoneal administration of either vehicle or a DHA nanoemulsion (nano-DHA) at 10, 50, or 250 μg/kg. Functional testing and histological analyses were performed at 28 days post-injury. The maximum intracavernosal pressure (ICP) and other measures of erectile function were significantly higher in the nano-DHA groups than in the vehicle group (*p* < 0.05). The ratio of area of expression of neuronal nitric oxide synthase (nNOS)/β-III tubulin, numbers of axon and smooth muscle cell content were significantly higher in the 50 μg/kg nano-DHA group than in the vehicle group (*p* < 0.05). A qualitative increase in the smooth muscle cells/collagen ratio and decrease in apoptosis was observed in the nano-DHA groups relative to the vehicle group: however, these differences were not statistically significant. Our data demonstrate that nano-DHA, particularly the 50 μg/kg regimen, improves erectile function after bilateral CN injury in rats by neuroprotection and other anti-fibrotic and anti-apoptotic mechanisms.

Radical prostatectomy is the first-line therapy for prostate cancer. Despite the introduction of improved surgical techniques and nerve-sparing procedures, erectile dysfunction observed after radical prostatectomy due to neuropraxia of the cavernous nerves (CNs) is common. According to recent estimates, normal erectile function following nerve-sparing radical prostatectomy is only 20–25%, and these rates have not improved over the last 17 years^1^. A majority of men lose erectile function for up to 3 months after surgery. Recovery often requires a minimum of 2 years, and full recovery of normal function is rare. Several therapeutic approaches including rehabilitation with PDE5 inhibitors, immunomodulation, neurotrophic factor administration, and regenerative techniques (e.g., stem cell therapy) either have failed to translate to clinical use or have not been studied in humans due to various concerns, such as use in cancer patients and unfavourable pharmacokinetics^2^.

Polyunsaturated fatty acids (PUFAs) are constituents of the cell membrane and the primary structural components of the brain, skin, sperm, testicles, and retina. *In vivo* and *in vitro* studies have characterized the neuroprotective effects of omega-3 PUFAs, particularly docosahexaenoic acid (DHA)^3^,^4^,^5^,^6^. Paterniti *et al.* demonstrated in a model of spinal cord injury that DHA treatment mediates an anti-inflammatory effect, attenuates the expression of inducible nitric oxide synthase (iNOS), interferes with neuronal apoptosis, and promotes motor recovery. The authors also demonstrated the efficacy of DHA in an *in vitro* model of dorsal root ganglion oxidative stress injury^7^.

It is well known that apoptosis, loss of smooth muscle cells, fibrosis, and abnormal neuronal nitric oxide synthase (nNOS) activity occur in response to CN injury^8^. Additionally, it has been suggested that PUFAs are capable of altering penile morphological features including the density of smooth muscle cells and collagen fibers^9^, which are often implicated in cavernous nerve injury. Therefore, DHA can be considered a candidate therapy for the treatment of erectile dysfunction following CN injury. However, the administration of DHA in high concentrations results in a loss of its beneficial actions^10^ and produces toxicity at concentrations >100 μg/mL^11^. Therefore, studies are required to characterize the therapeutic utility and associated therapeutic index of DHA for the treatment of erectile dysfunction due to CN injury. Here, we aimed to characterize the effect and appropriate dosage of nanoemulsion (nano)-DHA in a rat model of bilateral CN injury and erectile dysfunction. Specifically, we investigated the effect of 3 regimens of nano-DHA (10 μg/kg, 50 μg/kg, and 250 μg/kg) on functional and structural changes in the corpus cavernosum (CC) and CNs.

## Results

### Nano-DHA restores erectile function

**Figure 1:**
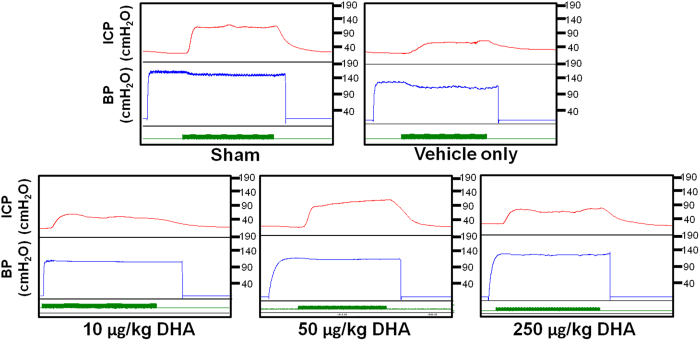
Recordings of intracavernosal pressure (ICP) and arterial blood pressure (BP) in the sham, vehicle, and nano-DHA-treated groups (10 μg/kg, 50 μg/kg, and 250 μg/kg, respectively) at 28 days post-injury. The x-axis represents time in seconds and the green bar represents an electrical stimulus of 60 s.

The effects of nano-DHA treatment on recovery of erectile function are illustrated in Fig. 1. It shows the intracavernosal pressure (ICP) and arterial blood pressure (BP) in the sham, vehicle, and nano-DHA-treated (10 μg/kg, 50 μg/kg, and 250 μg/kg, respectively) groups at 28 days post-injury. The maximum ICP of nano-DHA groups (10 μg/kg, 67.93 ± 20.72; 50 μg/kg, 94.05 ± 12.22; 250 μg/kg, 73.64 ± 10.08) and the sham group (105.92 ± 17.29) were significantly higher than those of the vehicle group (40.63 ± 9.87) (*p* < 0.05 for all comparisons). The area under the curve (AUC) for ICP and ∆ICP/MAP measures and the maximum ICP/MAP values were significantly higher for all nano-DHA groups (except the 10 μg/kg group) and the sham group than those for the vehicle group (*p* < 0.05 for all comparisons). Of note, the maximum ICP of the 50 μg/kg nano-DHA group was higher than that of the 10 μg/kg nano-DHA group (Table 1).

### Nano-DHA enhance the nNOS-positive fibers in the cavernous nerve and the dorsal penile nerve

**Figure 2:**
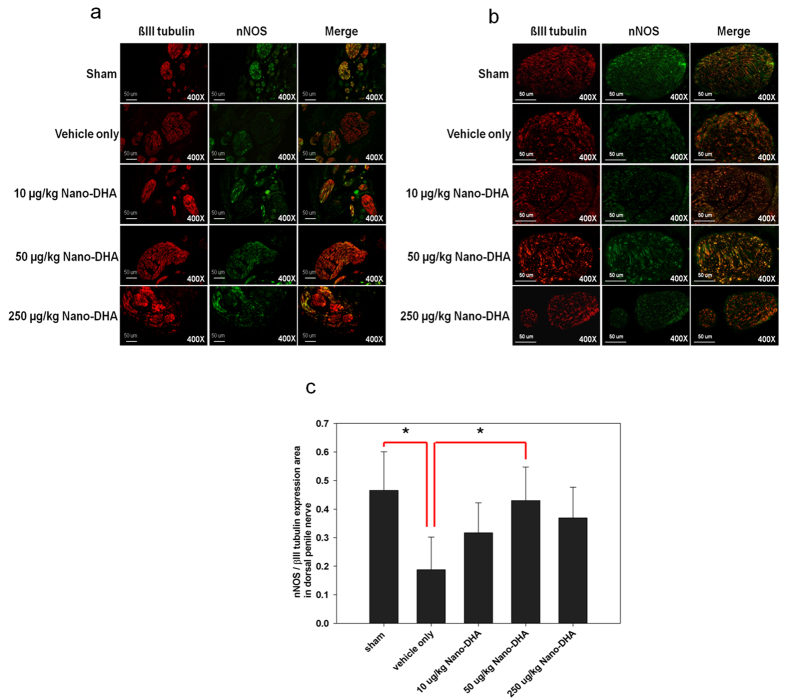
Immunofluorescence staining for nNOS in (**a**) the cavernous nerve (CN) fibre and (**b**) the dorsal penile nerve (DPN) of the sham, vehicle, and nano-DHA-treated groups at 28 days post-injury. Representative images for each group are shown (nNOS, green; β-III tubulin, red), original magnification 400x. (**c**) nNOS-positive nerve fibres in the DPN quantified as the area of nNOS-positive nerve fibres/β-III tubulin. Quantitative analysis showed that the number of nNOS-positive nerve fibres was increased in the nano-DHA-treated groups relative to the vehicle-treated group. *p < 0.05 versus vehicle group.

The nNOS-positive fibers of the CN and dorsal penile nerve (DPN) were immunostained for β-III-tubulin to identify nerve fibers positive for nNOS and to quantify their nNOS content (representative images of each group in Fig. 2a,b). The result shows that the number of nNOS-positive nerve fibres in the CN and DPN was increased in the nano-DHA-treated group relative to the vehicle-treated group. The ratio of the area of expression of nNOS/β-III tubulin was significantly higher in the 50 μg/kg nano-DHA group and the sham group than in the vehicle group (*p* < 0.05 for both comparisons) (Fig. 2c).

### Nano-DHA restores the axonal contents of the dorsal penile nerve

**Figure 3:**
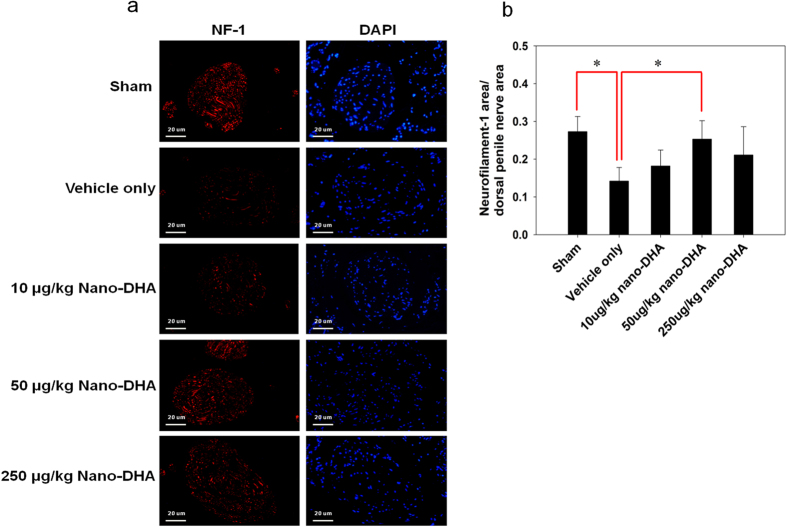
Immunofluorescence staining for neurofilament (NF)-1 expression in the dorsal penile nerve. (**a**) Representative images of the dorsal penile nerve from each group are shown. (NF-1, red; nuclear, blue; original magnification 400x). (**b**) Quantification of the NF-1 positive nerve fibers of the dorsal penile nerves expressed as the area of NF-1-positive nerve fiber/dorsal penile nerve. The quantitative analysis shows that the number of NF-1 positive nerve fibers is dramatically reduced at 28 d post-injury compared with the sham group. *p < 0.05 versus vehicle group.

The axon contents of the dorsal penile nerve were assessed for neurofilament-1 (NF-1) expression by immunofluorescence staining. Representative images of each group were shown in Fig. 3a. There was a significant reduction in NF-1 expression in the dorsal penile nerve in the vehicle only group compared with the 50 μg/kg nano-DHA group and sham group (*p* < 0.05; Fig. 3b). In additional, no significant correlation was uncovered between NF-1 expression and functional result.

### Nano-DHA decreases iNOS production in the dorsal penile nerve

**Figure 4:**
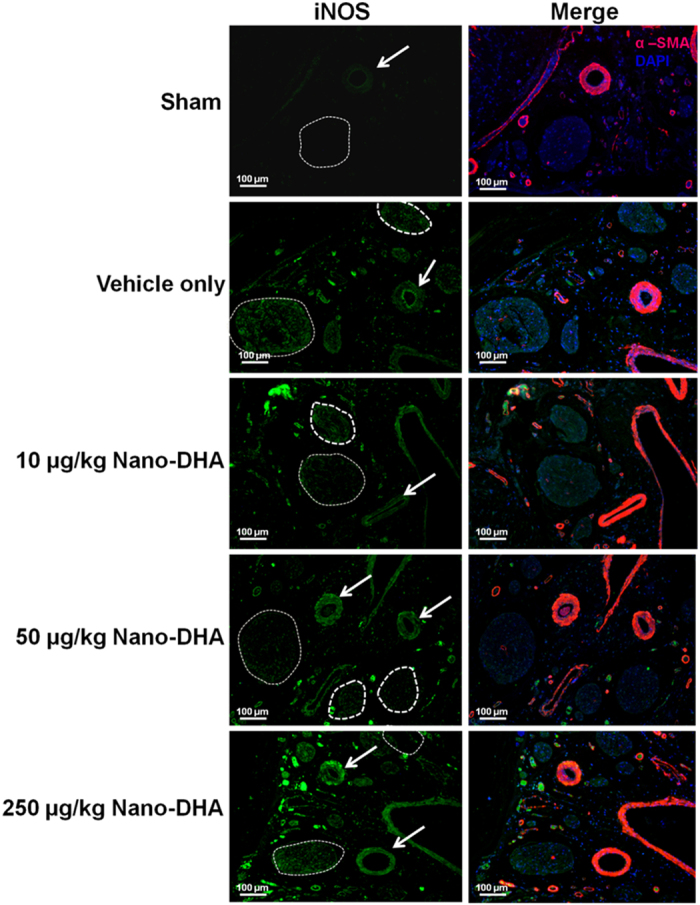
The distribution of inducible NOS (iNOS) in the dorsal penile nerve and artery bundles of cavernous nerve injury rat. Immunofluorescent double-staining of penile tissue performed with antibody to α-smooth muscle actin (α-SMA; an artery marker) and iNOS. (**a**) Representative fluorescence images of iNOS in the dorsal penile nerve and artery from each group are shown. (iNOS, green; smooth muscle, red; nuclear, blue; original magnification 100x). Note increase in iNOS expression in dorsal nerve bundle of vehicle only group. The dot circle is dorsal penile nerve; the arrow is dorsal artery. DAPI, 4,6-diamidino-2-phenylindole.

We performed immunofluorescence staining to evaluate the expression of iNOS in the dorsal penile nerve and artery bundle of penile tissue (Fig. 4). The result showed an increase in iNOS expression in both dorsal nerve bundle and the dorsal artery of CN injury rat compared with that in sham group, whereas iNOS expression in the dorsal penile nerve bundle were decreased in 50 μg/kg nano-DHA treatment group. However, there was no difference in the expression of iNOS of dorsal artery among all treated and vehicle only groups.

### Nano-DHA increases smooth muscle cell content and attenuates collagen deposition in the corpus cavernosum

**Figure 5:**
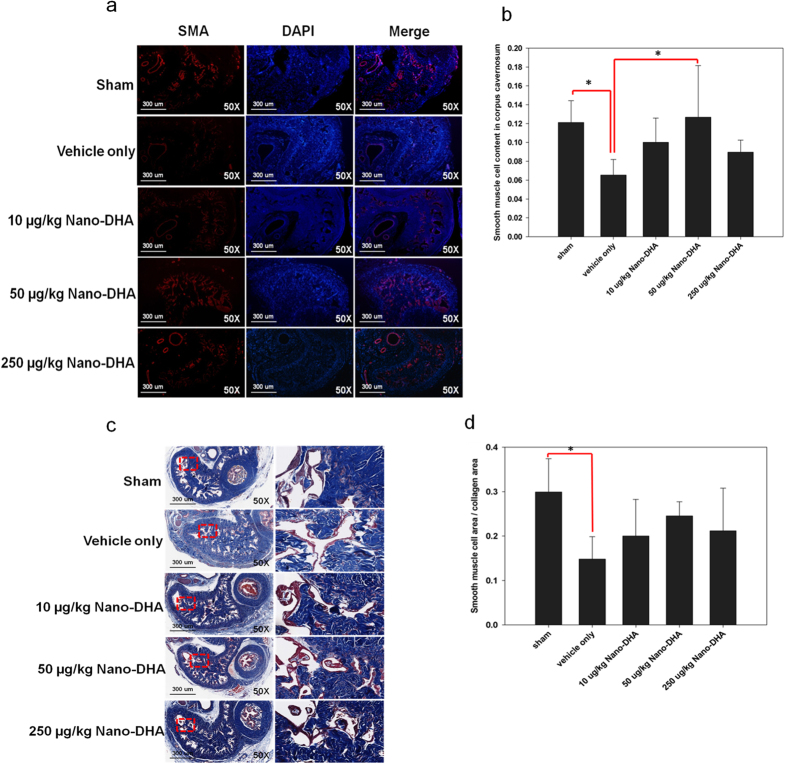
Histological analyses in the corpus cavernosum (CC) 28 days post-injury. (**a**) Representative fluorescence images of α-SMA-positive areas in the rat penile CC (smooth muscle, green; nuclei, blue), original magnification 50x. (**b**) SMC content in the CC quantified as the α-SMA-positive area/CC area. *p < 0.05 versus the vehicle group. (**c**) Masson’s trichrome staining for smooth muscle and collagen in the CC (smooth muscle cells, red; collagen, blue), original magnification 50x. The red squares show a higher magnification 400x. (**d**) Fibrosis severity quantified as the ratio of smooth muscle cell area and collagen area. *p < 0.05 versus the vehicle group.

Smooth muscle actin (α-SMA) was significantly higher in the 50 μg/kg nano-DHA group and the sham group than in the vehicle group (*p* < 0.05 for both comparisons). Masson’s Trichrome staining in the area of the CC lesion exhibited reduced collagen in the nano-DHA groups and the sham group relative to the vehicle only group (Fig. 5). The ratio of the area of expression of smooth muscle cells and collagen was higher in all nano-DHA groups and the sham group than in the vehicle group, signifying reduced fibrosis after treatment with nano-DHA; however, this difference was only statistically significant for the sham group (*p* < 0.05).

### Nano-DHA rescues cell damage in the corpus cavernosum

**Figure 6:**
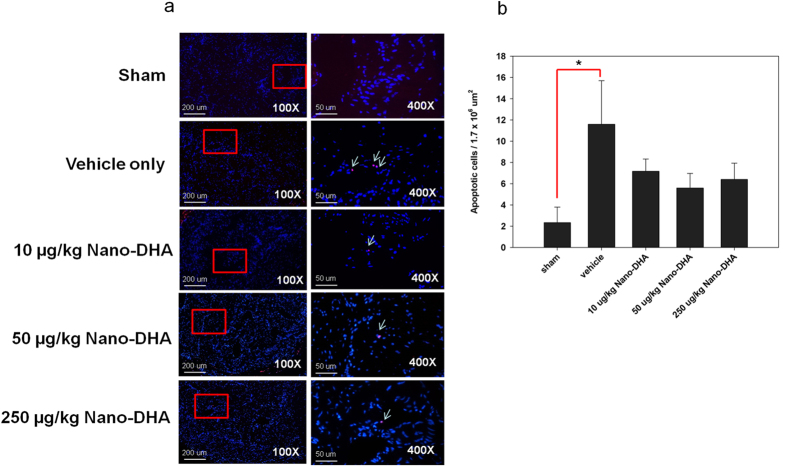
Transferase-mediated dUTP-biotin nick end labelling staining co-localization with nuclear 40,6-diamidino-2-phenylindole (DAPI) staining, original magnifications x100 and x400. Cells positive for both transferase-mediated dUTP-biotin nick end labelling and DAPI were considered to be apoptotic. Apoptosis was increased in the rat corpus cavernosum after cavernous nerve crush without. *p < 0.05 versus the vehicle group.

Figure 6 depicts an increase in apoptotic cells in the CC positive for both transferase-mediated dUTP-biotin nick end labelling (TUNEL) and 40,6-diamidino-2-phenylindole (DAPI) after CN nerve crush. In nano-DHA groups, the proportion of apoptotic cells per unit was lower but statistically insignificant from the vehicle group, whereas the sham group exhibited a significantly lower proportion of apoptotic cells per unit relative to the vehicle group (*p* < 0.05).

## Discussion

Despite the common use of radical prostatectomy for the treatment of prostate cancer, the preservation of penile erectile function remains a challenge due to mechanical damage to the CN. In the present study, we found that treatment with nano-DHA resulted in the recovery of erectile function as defined by increases in ICP, MAP, and other functional measures. The functional status in terms of maximum ICP/MAP following CN injury was 35% at 28 days post-injury in the vehicle group, which is consistent with previous studies in this model^8^,^12^. The maximum ICP/MAP was increased to 64% following injury in the 50 μg/kg nano-DHA group. Indeed, the therapeutic effect of nano-DHA was most prominent in the 50 μg/kg group (maximum ICP, 94.05 ± 12.22 cmH_2_O vs. 40.63 ± 9.87 cmH_2_O in the vehicle group; maximum ICP/MAP, 0.64 ± 0.18 vs. 0.35 ± 0.10 in the vehicle group). These data indicate that nano-DHA has therapeutic utility for promoting the functional recovery of erectile function following mechanical damage to the CN.

We examined whether functional recovery was accompanied by the improvement of neurological and histological markers of injury in our study. Both smooth muscle cell actin and collagen play an important role in fibroblast contractility^13^. The ratio of smooth muscle cells to collagen content is progressively reduced following CN injury or resection, indicating the increasing severity of fibrosis post-injury^8^. Indeed, bilateral CN ablation has been reported to induce fibrosis by reducing the number of smooth muscle cells and increasing collagen synthesis^14^. In the present study, we observed a decrease in the ratio of smooth muscle actin to collagen following CN injury, but only qualitative attenuation of this ratio after treatment with nano-DHA. It is possible that our results did not reach statistical significance due to the natural trend of increasing fibrosis after CN injury. A longer follow-up period may be required to observe statistically significant improvements in fibrosis. Nevertheless, these findings are consistent with the known anti-fibrotic properties of nutritional DHA^15^ and the attenuation of profibrotic factors such as transforming growth factor beta (TGFβ) and connective tissue growth factor (CTFG) following injury^16^,^17^.

Reductions of nNOS and nitric oxide (NO) required to sustain penile erection and increased apoptosis of penile smooth muscle cells are also implicated in erectile dysfunction following CN injury^18^,^19^. DHA is an important mediator of NOS activity and is able to modulate lipid peroxidation and protein oxidation to inhibit apoptosis^3^,^6^,^20^,^21^,^22^. Here, we showed that nano-DHA increased the expression of nNOS and β-III tubulin in the DPN and CN, indicative of nerve repair. iNOS is shown to modulate critical features of inflammation, neovascularization, and collagen deposition on the fibrovascular tissue induced by sponge implants in mice^23^ [42]. It has been also demonstrated to be upregulated with age in rat penis in association with ED^24^ [17]. Our results revealed that CN injury induces inflammation by increased the expression of iNOS in the dorsal penile nerve and artery. Nano-DHA treatment attenuates the expression of iNOS in dorsal penile nerve when compared with untreated injured rat. We clearly demonstrated that treatment with DHA could increase the rate of regeneration of axon and preservation of nNOS-positive nerve fibers by reducing the inflammatory response after nerve injury.

Furthermore, nano-DHA qualitatively decreased damage to the CC, as exhibited by the preservation of corporal smooth muscle cell and an apparent but not statistically significant decrease in the proportion of apoptotic cells following CN injury. Of note, while the TUNEL assay is highly sensitive, it has poor precision in detecting the magnitude of broken DNA strands, and does not adequately distinguish between cells undergoing apoptosis and cells in the processes of DNA repair^25^. We believe that this may explain the lack of statistically significant observations regarding apoptosis after nano-DHA administration in our study. The ability to protect neurons against cell death is critical to functional recovery following traumatic neuronal injury. Our findings provide evidence that nano-DHA has utility for penile rehabilitation via the regeneration of nNOS-containing nerves, increase of the axonal contents, reducing of neural inflammation and the reversal of smooth muscle cell apoptosis. Indeed, it has been reported that DHA may promote penile rehabilitation by stimulating neurite growth and synaptogenesis via the inhibition of nitric oxide production and the enhancement of glutamatergic transmission^11^,^25^,^26^.

DHA is a key component of neuronal membranes at sites of signal transduction, and is reduced following neuronal injury^27^. DHA treatment following various types of injuries such as traumatic brain injury^27^, spinal cord injury^3^,^5^,^6^, and ischemic stroke^28^ has led to neural recovery by reducing oxidative stress and attenuating inflammatory responses. There is evidence that DHA exerts its antioxidant and anti-inflammatory effect by suppressing pro-inflammatory cytokines such as tumour necrosis factor-α (TNF- α), decreasing nitrite levels, and promoting glutathione (GSH) and antioxidant enzyme activities.

In conclusion, nano-DHA (particularly the 50 μg/kg regimen) restored erectile function in a rat model of erectile dysfunction due to bilateral CN injury, and functional improvement was accompanied by structural recovery of the penile tissue. The underlying mechanism of nano-DHA in restoration of erectile function is neuroprotection and further contributed to anti-fibrotic and anti-apoptotic of corpus cavernosum. While additional studies are required to verify our findings, the documented safety and tolerability of DHA makes it an excellent candidate for clinical use in the treatment of erectile dysfunction following radical prostatectomy.

## Methods

### Animals and experimental design

Forty Sprague Dawley rats (12 weeks, 400–450 g) were obtained from Bioasco Taiwan (Taipei, Taiwan), housed in a controlled environment, and provided a standard diet *ad libitum*. Rats were randomly and equally divided into 5 groups (each group n = 8). CN injury or sham surgery was performed at the initiation of the study. Functional testing and histological analyses were performed in sham and CN injury groups at 28 days post-injury. After the functional evaluation, various tissues including the MPG, CN, CC, and DPN were collected for histological evaluation. All the animal experiment methods were conducted in accordance with the guidelines and regulation established by The Fu Jen Catholic University, Taiwan. All the experimental protocols in the present study were approved by The Fu Jen Catholic University Animal Care and Use Committee, Taiwan.

### Preparation of DHA nanoemulsion

The DHA nanoemulsion was prepared by combining 40 mg of oil containing 38 mg DHA/g with 1.2 g of Tween 80 and homogenizing with a glass rod. The homogenate was then diluted with 10.8 mL of deionized water to a total volume of 12 mL. The final mixture was sonicated for 1 h and contained a final concentration of 126.7 mg/L DHA.

### Surgical procedures

Rats were anesthetized with 50 mg/kg pentobarbital sodium and the abdomen was shaved and disinfected with an iodine-based solution. A lower midline abdominal incision was made and the prostate gland was exposed. The posterolateral CNs and MPG were identified bilaterally. At this point in the sham group, there was no further surgical manipulation. In the 4 CN injury groups, the CNs were isolated and a crushing injury was applied using a haemostat clamp (Roboz Surgical Instrument Co., Inc., Gaithersburg, MD, USA) for 2 min. Lastly, the 4 CN injury groups were administered vehicle (saline), 10 μg/Kg nano-DHA, 50 μg/Kg nano-DHA, or 250 μg/Kg nano-DHA intraperitoneally and the abdomen was closed in 2 layers.

### Measurement of erectile response

Erectile response was surgically assessed in all rats 4 weeks after sham or CN injury surgery and nano-DHA or vehicle injection. Rats were prepared for surgery as previously described and the abdomen was opened through a repeat midline abdominal incision. The CNs were exposed and isolated, and the crus of the penis was identified. A 24-gauge needle containing 50 U/mL heparin solution was inserted into the right penile crus and connected to a polyethylene-50 tube to measure intracavernosal pressure (ICP) with an MP36 pressure transducer (Biopac Systems Inc., CA, USA) and BSL 3.7.3 software (Biopac Systems). CNs were stimulated using a bipolar stainless steel electrode. Monophasic rectangular pulses were generated by a computer with a DS3 constant-current isolated stimulator (AutoMate Scientific Inc., CA, USA). The stimulus parameters were as follows: amplitude, 1.5 mA; frequency, 20 Hz; pulse duration, 0.2 ms; and total duration, 60 s. Erectile tissue response was determined in real time and measured by the maximal ICP, change in ICP (ΔICP), area under the ICP curve, and ratio of change in ICP and mean arterial pressure (MAP; ΔICP/MAP). The ICP operator was blinded to the treatment groups.

### Immunofluorescence staining

All animals were sacrificed by administration of a high dose of pentobarbital sodium solution. Tissue from the middle portion of the penis was collected from each of the 36 rats, fixed with formalin for 24 h (10% formaldehyde w/v), and subsequently dehydrated, post-fixed, and embedded in paraffin. Cross-sections of the embedded penile tissue were cut into 5-μm-thick slices, fixed, and deparaffinized in xylene for 10 min. The procedure was repeated twice for a total of 3 treatments and followed by hydration in graded alcohols. Slides were incubated for 1 h in 10% goat serum/2% bovine serum albumin/0.2% Triton X-100 (Sigma–Aldrich, St. Louis, MO, USA) at room temperature. The tissues were subsequently removed from the solutions and incubated overnight at 4 °C with the following primary antibodies: rabbit anti-neural nitric oxide synthase (nNOS; Santa Cruz Biotechnology, Santa Cruz, CA, USA), mouse anti–neuron-specific β-III tubulin, mouse anti-α-smooth muscle actin (Abcam Inc., Cambridge, MA, USA), rabbit anti- iNOS (Calbiochem, La Jolla, CA, USA) and mouse anti-neurofilament (NF-1) (Santa Cruz Biotechnology, Santa Cruz, California, USA). After incubation, the tissues were incubated with a 1:400 dilution of secondary antibody conjugated to Alexa Fluor 488 or Texas Red (Invitrogen, Carlsbad, CA, USA) for 1 h. Slides were then evaluated by fluorescence microscopy. For further analysis of nNOS and smooth muscle cell content, the ratio of the area of nNOS-positive cells to the area of β-III tubulin-positive cells in nerve fibres and the α-smooth muscle actin area of the CC were calculated at 400x and 100x magnifications, respectively. All computerized histomorphometric analyses were performed using ImageJ (National Institutes of Health, Bethesda, MD, USA).

### Masson’s Trichrome Staining

Penile tissue sections were fixed with 10% buffered formalin and stained using the Masson trichrome staining reagent kit according to the manufacturer’s instructions (Muto Pure Chemicals, Tokyo, Japan). All computerized histomorphometric analyses of the CC were performed using Olympus cellSens software (Olympus LATIN AMERICA, INC., Miami, FL, USA).

### Transferase-mediated dUTP-biotin nick end labelling (TUNEL) staining

The quantification of apoptotic cells was performed by detecting DNA damage *in-situ* with the Apo-BrdU *In Situ* DNA Fragmentation Assay Kit (BioVision, Inc., CA, USA) and counterstaining with 4′,6-diamidino-2-phenylindole (DAPI) in paraffin-embedded tissue sections. The apoptotic index was expressed as the number of TUNEL and DAPI-positive cells in 6 randomly chosen high-power fields (×400) of the sinusoids in corpus cavernosum per rat, which were photographed and digitally analyzed.

### Statistical Analysis

The overall data were summarized using descriptive statistics and expressed as the mean ± standard deviation. Comparison of multiple treatment groups was performed with a 1-way analysis of variance and pairwise post hoc comparisons with the Scheffe test. All statistical analyses were performed using SPSS Version 12.0 (SPSS Inc., Chicago, IL, USA) for Windows and *p* < 0.05 was considered to be statistically significant.

## Additional Information

**How to cite this article**: Liao, C.-H. *et al.* Neuroprotective effect of docosahexaenoic acid nanoemulsion on erectile function in a rat model of bilateral cavernous nerve injury. *Sci. Rep.* **6**, 33040; doi: 10.1038/srep33040 (2016).

## Figures and Tables

**Table 1:** Intracavernous and mean arterial pressure measurements following cavernous nerve electrostimulation.

Groups	Maximum ICP	∆ICP	AUC	∆ICP/MAP	Max ICP/MAP
Sham	105.92 ± 17.29*	87.04 ± 9.52*	4333.12 ± 948.81*	0.63 ± 0.10*	0.76 ± 0.08*
Vehicle only	40.63 ± 9.87	22.15 ± 8.69	653.91 ± 253.55	0.20 ± 0.09	0.35 ± 0.10
10 μg/Kg nano-DHA	67.93 ± 20.72**	49.43 ± 20.32*	2139.55 ± 1338.35	0.36 ± 0.13	0.48 ± 0.13
50 μg/Kg nano-DHA	94.05 ± 12.22*	71.31 ± 17.77*	3068.14 ± 1211.60*	0.50 ± 0.20*	0.64 ± 0.18*
250 μg/Kg nano-DHA	73.64 ± 10.08*	56.23 ± 12.80*	2405 ± 633.35*	0.44 ± 0.09*	0.59 ± 0.06*

AUC, area under the curve; ICP, intracavernous pressure; MAP, mean arterial pressure.

∆ICP = Max ICP − Min ICP.

*p < 0.05 versus vehicle only group.

**p < 0.05 versus 50 μg/kg nano-DHA group.

## References

[b1] Schauer I., Keller E., Müller A. & Madersbacher S. Have rates of erectile dysfunction improved within the past 17 years after radical analysis of the control arms of prospective randomized prostatectomy? A systematic trials on penile rehabilitation. Andrology 3, 661–665 (2015). 26198796 10.1111/andr.12060

[b2] Weyne E., Castiglione F., Van der Aa F., Bivalacqua T. J. & Albersen M. Landmarks in erectile function recovery after radical prostatectomy. Nat. Rev. Urol. 12, 289–297 (2015). 25868558 10.1038/nrurol.2015.72

[b3] Paterniti I. *et al.* Docosahexaenoic acid attenuates the early inflammatory response following spinal cord injury in mice: *in-vivo* and *in-vitro* studies. J. Neuroinflammation 11, 6 (2014). 24405628 10.1186/1742-2094-11-6 PMC3895696

[b4] Serini S. & Calviello G. Reduction of oxidative/nitrosative stress in brain and its involvement in the neuroprotective effect of n-3 PUFA in Alzheimer’s disease. Curr. Alzheimer Res. 13, 123–134 (2016). 26391044 10.2174/1567205012666150921101147

[b5] Hall J. C., Priestley J. V., Perry V. H. & Michael-Titus A. T. Docosahexaenoic acid, but not eicosapentaenoic acid, reduces the early inflammatory response following compression spinal cord injury in the rat. J. Neurochem. 121, 738–750 (2012). 22404382 10.1111/j.1471-4159.2012.07726.x

[b6] Huang W. L. *et al.* A combination of intravenous and dietary docosahexaenoic acid significantly improves outcome after spinal cord injury. Brain 130, 3004–3019 (2007). 17901087 10.1093/brain/awm223

[b7] Paterniti I. *et al.* Docosahexaenoic acid attenuates the early inflammatory response following spinal cord injury in mice: *in-vivo* and *in-vitro* studies. J Neuroinflammation 11, 6 (2014). 24405628 10.1186/1742-2094-11-6 PMC3895696

[b8] Kim T. B., Cho M. C., Paick J. & Kim S. W. Is It Possible to Recover Erectile Function Spontaneously after Cavernous Nerve Injury? Time-Dependent Structural and Functional Changes in Corpus Cavernosum Following Cavernous Nerve Injury in Rats. Korean J. Androl. 30, 31 (2012).

[b9] Medeiros Júnior J. L. *et al.* Lard and/or canola oil-rich diets induce penile morphological alterations in a rat model. Acta. Cir. Bras. 29, 39–44 (2014). 25185055 10.1590/s0102-86502014001300008

[b10] Cha M. C., Meckling K. A. & Stewart C. Dietary docosahexaenoic acid levels influence the outcome of arabinosylcytosine chemotherapy in L1210 leukemic mice. Nutr. Cancer 44, 176–181 (2002). 12734065 10.1207/S15327914NC4402_09

[b11] Wang X. Neuroprotective effect of docosahexaenoic acid on glutamate-induced cytotoxicity in rat hippocampal cultures. Neuroreport 14, 2457–2461 (2003). 14663210 10.1097/00001756-200312190-00033

[b12] Mullerad M., Donohue J. F., Li P. S., Scardino P. T. & Mulhall J. P. Functional sequelae of cavernous nerve injury in the rat: is there model dependency. J. Sex. Med. 3, 77–83 (2006). 16409220 10.1111/j.1743-6109.2005.00158.x

[b13] Hinz B., Celetta G., Tomasek J. J., Gabbiani G. & Chaponnier C. Alpha-smooth muscle actin expression upregulates fibroblast contractile activity. Mol. Biol. Cell 12, 2730–2741 (2001). 11553712 10.1091/mbc.12.9.2730 PMC59708

[b14] Hu W. L. *et al.* Fibrosis of corpus cavernosum in animals following cavernous nerve ablation. Asian J. Androl. 6, 111–116 (2004). 15154084

[b15] Velten M., Britt R. D. Jr., Heyob K. M., Tipple T. E. & Rogers L. K. Maternal dietary docosahexaenoic acid supplementation attenuates fetal growth restriction and enhances pulmonary function in a newborn mouse model of perinatal inflammation. J. Nutr. 144, 258–266 (2014). 24453131 10.3945/jn.113.179259 PMC3927543

[b16] Miyazaki M. *et al.* Dietary docosahexaenoic acid ameliorates, but rapeseed oil and safflower oil accelerate renal injury in stroke-prone spontaneously hypertensive rats as compared with soybean oil, which is associated with expression for renal transforming growth factor-beta, fibronectin and renin. Biochim. Biophys. Acta. 1483, 101–110 (2000). 10601699 10.1016/s1388-1981(99)00180-8

[b17] Priante G., Musacchio E., Valvason C. & Baggio B. EPA and DHA suppress AngII- and arachidonic acid-induced expression of profibrotic genes in human mesangial cells. J. Nephrol. 22, 137–143 (2009). 19229829

[b18] Carrier S. *et al.* Regeneration of nitric oxide synthase-containing nerves after cavernous nerve neurotomy in the rat. J. Urol. 153, 1722–1727 (1995). 7536273

[b19] User H. M., Hairston J. H., Zelner D. J., McKenna K. E. & McVary K. T. Penile weight and cell subtype specific changes in a post-radical prostatectomy model of erectile dysfunction. J. Urol. 169, 1175–1179 (2003). 12576876 10.1097/01.ju.0000048974.47461.50

[b20] Engström K., Saldeen A. S., Yang B., Mehta J. L. & Saldeen T. Effect of fish oils containing different amounts of EPA, DHA, and antioxidants on plasma and brain fatty acids and brain nitric oxide synthase activity in rats. Ups. J. Med. Sci. 114, 206–213 (2009). 19961266 10.3109/03009730903268958 PMC2852776

[b21] Kim Y. J. & Chung H. Y. Antioxidative and anti-inflammatory actions of docosahexaenoic acid and eicosapentaenoic acid in renal epithelial cells and macrophages. J. Med. Food 10, 225–231 (2007). 17651056 10.1089/jmf.2006.092

[b22] Lu D. Y., Tsao Y. Y., Leung Y. M. & Su K. P. Docosahexaenoic acid suppresses neuroinflammatory responses and induces heme oxygenase-1 expression in BV-2 microglia: implications of antidepressant effects for ω-3 fatty acids. Neuropsychopharmacology 35, 2238–2248 (2010). 20668435 10.1038/npp.2010.98 PMC3055314

[b23] Cassini-Vieira P. *et al.* iNOS Activity Modulates Inflammation, Angiogenesis, and Tissue Fibrosis in Polyether-Polyurethane Synthetic Implants. Mediators Inflamm 2015, 138461 (2015). 10.1155/2015/138461 PMC4461775 26106257

[b24] Ferrini M., Magee T. R., Vernet D., Rajfer J. & González-Cadavid N. F. Aging-related expression of inducible nitric oxide synthase and markers of tissue damage in the rat penis. Biol Reprod. 64, 974–982 (2001). 11207215 10.1095/biolreprod64.3.974

[b25] Elmore S. Apoptosis: A review of programmed cell death. Toxicol. Pathol. 35, 495–516 (2007). 17562483 10.1080/01926230701320337 PMC2117903

[b26] Kim H. Y., Spector A. A. & Xiong Z. M. A synaptogenic amide N-docosahexaenoylethanolamide promotes hippocampal development. Prostaglandins Other Lipid Mediat. 96, 114–120 (2011). 21810478 10.1016/j.prostaglandins.2011.07.002 PMC3215906

[b27] Wu A., Ying Z. & Gomez-Pinilla F. Dietary strategy to repair plasma membrane after brain trauma: implications for plasticity and cognition. Neurorehabil. Neural Repair 28, 75–84 (2014). 23911971 10.1177/1545968313498650

[b28] Eady T. N. *et al.* Docosahexaenoic acid signaling modulates cell survival in experimental ischemic stroke penumbra and initiates long-term repair in young and aged rats. Plos One 7, e46151 (2012). 23118851 10.1371/journal.pone.0046151 PMC3484151

